# Evaluation of cellulose degrading bacteria isolated from the gut-system of cotton bollworm, *Helicoverpa armigera* and their potential values in biomass conversion

**DOI:** 10.7717/peerj.11254

**Published:** 2021-05-04

**Authors:** Mudasir A. Dar, Afrin F. Shaikh, Kiran D. Pawar, Rongrong Xie, Jianzhong Sun, Sabariswaran Kandasamy, Radhakrishna S. Pandit

**Affiliations:** 1 Biofuels Institute, School of the Environment and Safety Engineering, Jiangsu University, Zhenjiang, Jiangsu Province, China; 2 Department of Zoology, Savitribai Phule Pune University, Ganeshkhind, Pune, Maharashtra, India; 3 School of Nanoscience and Biotechnology, Shivaji University, Kolhapur, Maharashtra, India; 4 Institute of Energy Research, Jiangsu University, Zhenjiang, Jiangsu Province, China

**Keywords:** Lignocellulose, *Helicoverpa armigera*, Gut-regions, Cellulose degrading bacteria, Culture-dependent, Agro-waste

## Abstract

**Background:**

Cotton bollworm, *Helicoverpa armigera* is a widely distributed, devastating pest of over 200 crop plants that mainly consist of some cellulosic materials. Despite its economic importance as a pest, little is known about the diversity and community structure of gut symbiotic bacteria potentially functioned in cellulose digestion in different gut-sections of *H. armigera*. In view of this lacuna, we attempted to evaluate and characterize cellulose-degrading bacteria (CDB) from foregut, midgut, and hindgut -regions of *H. armigera* by using a culture-dependent approach.

**Methodology:**

The symbiotic bacteria were isolated from different gut-systems of *H. armigera* by enrichment techniques using Carboxymethyl cellulose sodium salt (CMC) as carbon source. The isolated bacteria were purified and subsequently screened for cellulose-degradation by plate-based method to display the zones of CMC clearance around the colonies. The identification and phylogeny of the gut-bacteria were reconstructed by using 16S rRNA gene sequencing. Different enzymes such as endoglucanase, exoglucanase, *β*-glucosidase, and xylanase were assayed to determine the cellulolytic repertoire of the isolated bacteria.

**Results:**

The enrichment of CDB and subsequent plate based screening methods resulted in isolation of 71 bacteria among which 54% of the bacteria were obtained from foregut. Among the isolated bacteria, 25 isolates showed discernible cellulose-degradation potential on CMC-agar plates. The phylogenetic analysis based on 16S rRNA gene amplification and sequencing affiliated these cellulolytic bacteria to two major phyla viz., Firmicutes and Proteobacteria. The members of the genus *Klebsiella* accounted for 39.43% of the total isolated bacteria while 31% of the *Bacillus* strains were enriched from hindgut region. The principal component analysis (PCA) further suggested that the members of *Bacillus* and *Klebsiella* together dominated the foregut and hindgut regions as they accounted for 68% of the total CDB. The four potential isolates selected on the basis of plate-based activities were further evaluated for their lignocellulases production by using various agricultural wastes as substrates. The PCA of the enzyme activities demonstrated that potential isolates majorly secreted endoglucanase and xylanase enzymes. Among the agro-wastes, multivariate analysis validated wheat husk (WH) and sugarcane bagasse (SCB) as most favorable substrates for xylanase and endoglucanase productions respectively. The overall findings suggest that *H. armigera* harbors diverse bacterial communities in different gut-sections that could assist the host in digestion processes, which may potentially serve as a valuable reservoir of some unique symbionts applied for biomass conversion in biofuel industry.

## Introduction

Insects represent the most diverse and dominant group of animals on earth whose successful survival relies largely on superior adaptations to varied environments alongside food resources. To achieve this success, insects have evolved sophisticated and highly specialized gut-systems to digest a variety of foods, predominantly lignocellulose which is dry matter of plant cell walls (PCW). These natural bomass utliziation systems (NBUS) possess extra-ordinary efficiency to metabolize different components of the lignocellulose (Sun & Scharf, 2010; Sun, Ding & Doran-Peterson, 2014). Due to the extraordinary digestion of lignocellulose, the insect-gut systems have been designated as world’s smallest natural bioreactors (Breznak & Brune, 1994). The potential digestion of lignocellulose is catalyzed by complex of enzymes called cellulases and hemicellulases. Cellulases and hemicellulases are the key players to hydrolyze cellulose and hemicellulose respectively (Tsegaye, Balomajumder & Roy, 2019a), which together comprise about 70% of the PCW contents. The principle enzymes of this complex are endo-*β*-1, 4-glucanases, exo-*β*-1, 4-glucanases (Avicellase) and *β*-1, 4-glucosidase (Wilson & Irwin, 1999). The endoglucanase randomly attacks the *β*-1, 4 bonds of inner chains in cellulose thereby breaking the large molecules into shorter stretches which are then targeted by exoglucanase that releases the terminal disaccharide units called cellobiose. The *β*-glucosidase finally breaks the cellobiose into its monomeric sugar residues which can be assimilated through different metabolic pathways. Similarly, xylanases are the enzyme complexes that degrade hemicellulose contents. Hitherto, only few of the insect groups like termites and beetles are known to secret their endogenous cellulases (Watanabe & Tokuda, 2001). However, these insects possess very few cellulase encoding genes in their genomes when compared with symbiotic gut bacteria. Consequently, the endogenous cellulases does not perform complete degradation of cellulose (Ulyshen, 2016). Thus, majority of the insects including termites and beetles still rely on symbiosis with gut-microorganisms such as bacteria or protozoa, for this repertoire (Xie et al., 2014). Due to this emblematic symbiosis, most of the termites achieve efficient digestion of lignocellulose (60–90%) with significant contribution from gut bacteria (Calusinka et al., 2020). Moreover, the fundamental structure of insect guts having enormous surface-to volume ratio is shaped to shelter a diversity of bacteria ((Brune & Carsten, 2015). To date many potential lignocellulose hydrolyzing bacteria from Firmicutes and Proteobacteria have been isolated from termites (Tsegaye, Balomajumder & Roy, 2018a; Tsegaye, Balomajumder & Roy, 2018b; Tsegaye, Balomajumder & Roy, 2019b; Auer et al., 2017).

During the course of evolution, insects have developed an intricate relationship with gut symbiotic bacteria, that contribute to their physiology as well as development *(Engel & Moran, 2013) .* The gut bacteria present the metabolic properties that are usually absent in hosts, thus, function as ‘microbial brokers’ enabling the polyphagous insects to overcome the barriers of herbivory (Dillon & Dillon, 2004). The gut bacteria are known to augment digestion of food, besides providing essential vitamins to the insects. The symbiotic bacteria attribute the host with a suite of enzymes that hydrolyze lignocellulose into readily usable metabolites thereby, contribute to the metabolism and energy requirements of the host *(Brune, Miambi & Breznak, 1995)*. Hitherto, the process of symbiosis has been well studied in termites and beetles, however, very little is known about the association of gut bacteria in Lepidopteran insects particularly with respect to cellulose digestion. The order Lepidoptera containing moths and butterflies are among the highly diversified insects that are exclusively phytophagous. They are considered as the most devastating agricultural pests worldwide (*Sree & Varma, 2015*). Yet, clear evidence for bacterial associates playing a fundamental role in lepidopteran biology is scarce. Moreover, out of 180,000 recognized lepidopteran species, only <0.1% of species have been studied for host-bacterial symbiosis (Mitter, Davis & Cummings, 2017). This in other words indicates that our knowledge about bacterial associates in Lepidoptera is still very limited. A comprehensive understanding of the functions of gut bacterial symbionts in Lepidoptera will not only usher the current practices of integrated pest management (IPM) but would also help to understand the vital features of gut bacterial symbionts towards the adaptation of lepidopteran larvae to different diets and habitats.

Among different species of polyphagous Lepidoptera, the cotton bollworm, *Helicoverpa armigera* inhabits diverse niches and is the non-host specific pest of over 200 commercial plant species. It is a prolific pest, cosmopolitan in distribution widely occurring in the developing world where it causes heavy yield losses to a diverse range of dicots like cotton, tomato, chickpea, potato, brinjal and monocot crops such as maize, sorghum, bajra (Fitt, 1989), etc. The *H. armigera  * is generally considered as a serious pest due to its ability to develop resistance to insecticides, broader host range, and persistence in cropping areas. Although there have been numerous reports on cellulolytic activity in insects like termites (Geng et al., 2018; Tokuda et al., 1997) and beetles (Luo et al., 2018; Lemke et al., 2003), the relevant information for the cellulolytic microbes in Lepidoptera *(Duplouy & Hornett, 2018)* particularly *H. armigera* is scanty. The gut-microbiome prospection of *H. armigera* would not only contribute to basic understanding of the pest-biology, host-microbiome co-evolution but would also highlight its prospective applications for discovery and bioengineering of digestive enzymes, for use in biorefinery and paper or pulp industry alongside biofuel production.

The gut-systems of insect are structurally complex divisible into three major regions, viz., foregut (FG), midgut (MG), and hindgut (HG), each possessing unique biotic and abiotic features. In insects, the gut-regions such as FG, MG and HG are considered as specialized compartments with distinctive functionalities. Similarly, the gut-systems of *H. armigera* being different in origin and structure, we hypothesized that each of the gut sections might foster a unique set of bacterial community. In light of this objective, the cellulolytic bacteria inhabiting different gut-regions of *H. armigera* were explored by using enrichment technique followed by 16S rRNA gene amplification and sequencing. Additionally, the valuable bacteria performed in biomass conversion were selected and evaluated for their potentials in producing lignocellulose hydrolyzing enzymes that can break down various agricultural wastes, such as sugarcane bagasse (SCB), grass straw (GS), wheat husk (WH), etc.

## Materials and Methods

### Enrichment and isolation of bacteria

**Figure 1:**
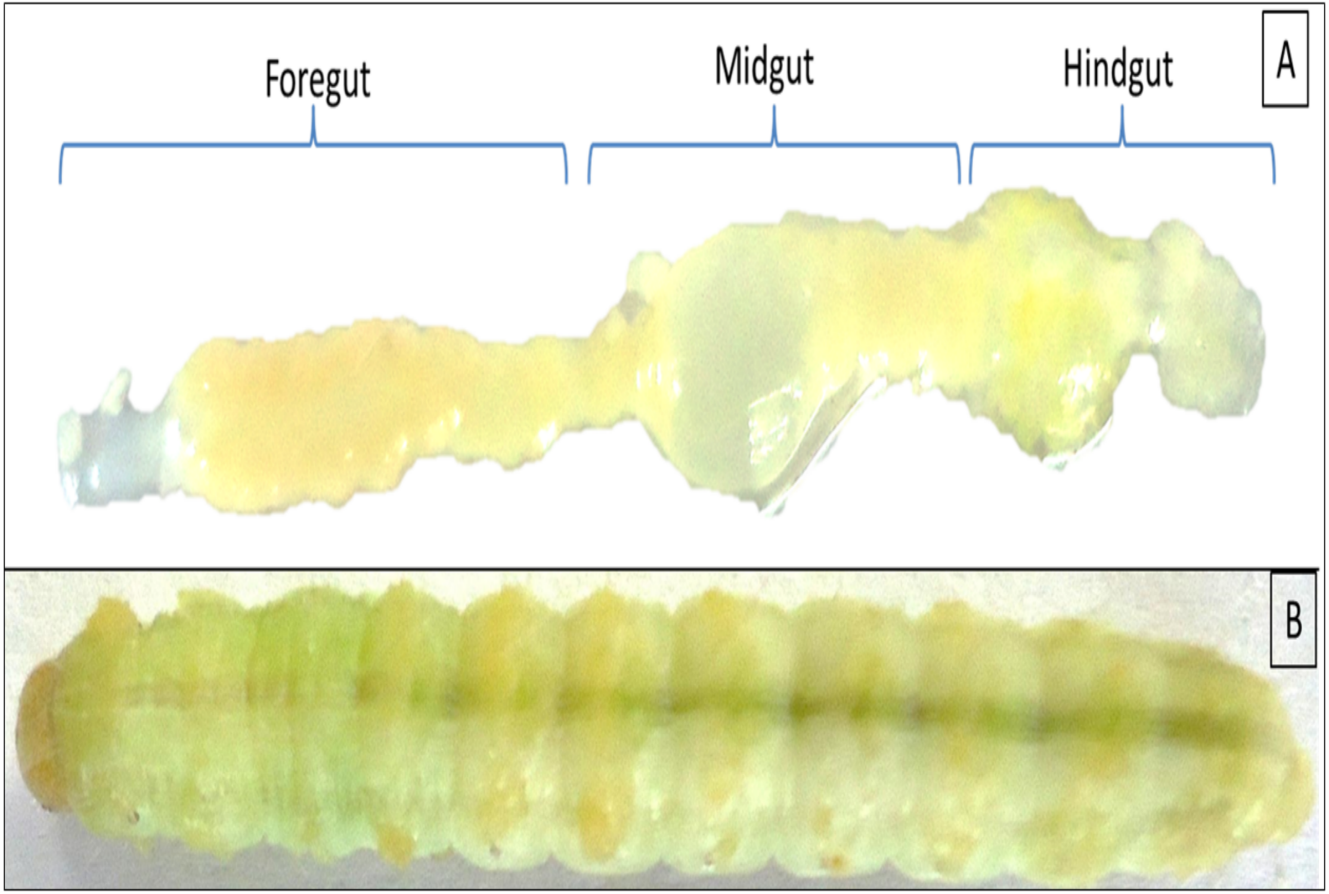
Digestive system of *Helicoverpa armigera* showing foregut, midgut and hindgut regions (A) excluding mouth and anus. (B) 5th instar larvae of *H. armigera*.

Twenty individuals of late 5th instar larvae of *H. armigera* were collected from the pea crops of Saswad area (18.55°N 74.00°E in Pune, Maharashtra), India. The co-author Ms. Afrin F. Shaikh had good contact with local farmers, Abdul Nabi ChandSaheb Shaikh and Mane Gajanan, who allowed us to visit their fields and collect insect samples. The collected larvae were starved overnight, surface sterilized with 50 and 70% alcohol grades for 30 s each followed by a final wash with absolute ethanol for 1 min. The larvae were sacrificed under aseptic conditions in a biosafety hood to reveal the gut regions as mentioned previously (Dar et al., 2018). The gut systems were divided into FG-, MG- and HG- regions as shown in Fig. 1 with the aid of a sterilized magnifying lens (10X). The respective gut regions were weighed and homogenized in micro centrifuge tubes (MCT) containing 0.5 mL phosphate buffer saline (PBS pH 7.4). For enrichment of CDB community, the macerated gut regions were then separately inoculated to flasks containing Berg minimal salt medium (BMS) and 0.5% (w/v) CarboxyMethyl cellulose (CMC) sodium salt. The enrichment was carried out at 37 °C on an orbital shaker rotating at 150 rpm. On alternate days, aliquot ( one mL) of each culture was transferred to nine mL of freshly prepared BMS-CMC medium and incubated at 150 rpm, 37 °C. The remaining nine mL of enrichment culture was harvested to calculate total viable count (TVC) by spread plate technique on BMS-CMC-Agar plates. After discernible growth of isolates, the unique colonies with distinct morphologies were purified by repeatedly streaking on Luria Bertani (LB) as well as CMC-agar plates. The colonies were codified and screened further for CMCase activity by using BMS-CMC agar plates.

### Screening of cellulolytic bacteria

The screening of the isolated bacteria was performed as described previously (Dar, Pawar & Pandit, 2018) with Gram’s Iodine reagent (Kasana et al., 2008). The activity was determined based on the zone of clearance around the colonies in centimeters as low (1.0–2.0 cm), medium (2.1–3.0 cm) and high (above three cm). The isolates showing zone of clearance less than 0.5 cm in diameter were neglected to avoid the confusion with cellulose positive bacteria. The hydrolytic capacity (HC) of the CDB was also determined by calculating the ratio of clearance zone to the colony diameter (Hendricks, Doyle & Hugley, 1995). Subsequently, the isolates that exhibited potential activities were selected and further characterized for production of various enzymes as described below in the section, “Enzyme activities and principal component analysis”.

### Identification and phylogenetic analysis

The identification of cellulose degrading isolates were confirmed by 16S rRNA gene amplification and sequencing. To this end, gDNAs were extracted from overnight grown CDB by using HipurA™ soil DNA purification kit according to the manufacturer’s instructions. The purity of the extracted gDNA was confirmed by electrophoresis on 0.8% (w/v) Agarose gels while the quantification was carried out on a Biospectrometer^®^ nanodrop machine (Eppendorf India Pvt. Ltd.). The polymerase chain reaction (PCR) for the amplification of 16S rDNA was accomplished with bacteria-specific primers viz., 27F and 1492R (Weisburg et al., 1991). The 16S rRNA gene from each CDB was amplified in 50 µL PCR reactions that contained 25 µL *Taq* PCR Master Mix (Qiagen Pvt. Ltd.), 2.5 µL each of forward and reverse primers (10 pmol/l), 14 µL double distilled water and 6 µL of template DNA (10 ng/µL). The thermal cycling conditions employed were as: initial denaturation at 94  °C for 5 min, 30 cycles of denaturation at 94  °C, primer annealing at 55 °C and amplicon extension at 72 °C, each for 1 min, followed by a final extension at 72 °C for 7 min. The amplified 16S rRNA gene products were checked for purity on 1.2% (w/v) Agarose gels and then sequenced by using BigDye terminator cycle sequencing kit version 3.1 (Applied Biosystems, USA) using manufacturer’s instructions. The obtained sequences were analyzed in ChromasPro software and contigs prepared were submitted to NCBI BLASTn and GenBank for affiliation of closely related bacteria as well as accession numbers. The closely related 16S rRNA gene sequences retrieved from GenBank were aligned with 16S rRNA gene sequences of cellulolytic bacteria by using Clustal X program (Thompson et al., 1997). Lastly, the phylogenetic tree was reconstructed by Neighbor-Joining (NJ) method with Kimura 2 model using 1000 bootstrap replicates in MEGA 6.0 package(Tamura et al., 2013). Moreover, a principal component analysis (PCA) was carried out in PAST software v4.0 (Hammer, Harper & Ryan, 2001) to find the similarities and species-wise distribution of cellulase positive bacteria in 3 regions (viz., foregut, midgut and hindgut) of the insect gut.

### Optimization of substrate concentration and pH

To evaluate the effect of substrate concentration on cellulolytic activity of bacteria, individual bacterial isolates were cultured axenically on CMC-agar plates containing CMC in the range of 0.5 to 2.5%. After incubation at 37 °C for 24–48 h, the plates were stained with Gram’s iodine solution to perceive the zone of substrate clearance as a mark of cellulolytic activities. The diameter of the clearance zones were measured and used to calculate the hydrolytic capacity ratio. Similar to the effect of substrate concentration, optimization of pH was also carried out. To this end, the bacterial isolates were patched on BMS-CMC Agar plates having different pH ranging between pH 5.0 to 9.0.

### Growth curve determination

The growth patterns of CDB that showed higher activities were determined in BMS liquid media containing 0.5% CMC (w/v). The freshly prepared BMS medium was seeded with a single colony of individual bacterial isolates followed by incubation at 37  °C in a rotary incubator agitating with a speed of 160 rpm. The sample OD was measured at 600 nm after every 8 hrs till the bacteria showed declined growth and a curve of observed absorbance was plotted against time.

### Enzyme activities and principal component analysis

In the present study, the potential of selected CDB to produce different cellulolytic enzyme activities such as endoglucanase, exoglucanase, xylanase, and *β*-glucosidase were assessed by growing them on different agricultural wastes and commercially available LC sources as substrates. To accomplish this, the agricultural wastes such as sawdust (SD), GS, CS, SCB, WH and commercially available LC sources like filter paper (FP), CMC, xylan, etc were used. To determine enzyme activities, selected potential CDB were inoculated individually to BMS media with 1% of LC substrates, incubated by shaking at 150 rpm, 37  °C for 10 days. After the incubation period, samples were collected and centrifuged at 10,000 rpm for 10 min to harvest the supernatants that were treated as crude enzyme extracts for estimation of different enzyme activities. Enzyme activities like endoglucanase (CMCase), exoglucanase (Avicellase) and xylanase were determined by 3, 5 dinitrosalicylic acid (DNSA) method (Miller, 1959) following previously optimized protocols (Dar et al., 2015). The reducing sugars were measured at 550 nm in a Multi-Ex spectrophotometer (Thermo Scientific, Finland) using glucose and D-xylose as standards. However, the *β*–glucosidase activity was estimated by incubating a reaction mixture comprising one mL of 0.2% cellobiose (w/v) with one mL of enzyme extract at 40 ^∘^C for 1 hr in a water bath. The reactions were terminated by the addition of three mL DNSA reagent, followed by heating in boiling water for 5 min and recording the absorbances at 540 nm in a spectrophotometer. To determine the cumulative enzyme activity, sample aliquots obtained from selected bacteria were pooled together and centrifuged with 12,000 rpm for 10 min at 4 °C. The supernatants collected were subjected to above stated enzyme assays. The protein contents of the samples were determined by the method of Lowry et al. (1951) at 660 nm using bovine serum albumin (BSA) as standard. The enzyme activities are expressed as international unit (IU) where 1IU of activity is the amount of protein required to release 1 µMol of glucose equivalents under standard assay conditions.

In order to assess how the individual selected bacterial isolates produce different enzymes and establish the correlation between substrates and enzyme activities secreted by CDB, a multivariate PCA was employed. The PCA analyses were performed for individual potential isolates by using PAST software v4.0 (Hammer, Harper & Ryan, 2001). Then principal components were extracted by setting an Eigenvalue above 1 as threshold and biplots were reconstructed to determine the original variables and transformed observations onto the plane spanned by primary two components. The graphical visualization was carried out by using biplots to highlight the relationship among tested enzymes and relative substrates of individual bacteria.

### Statistical analysis

The data were analyzed statistically wherever required by using PAST software (version 4.0) and Microsoft office excel 2016. The graphs were plotted by using Microsoft office suite (version 2016) as well as Origin Lab., software version 8.5 except the PCA biplots which were drawn in PAST software. The experiments were triplicated and the observations are presented as means with standard deviations of independent replicates.

## Results

### Total viable count and screening of cellulose-degrading bacteria

**Figure 2:**
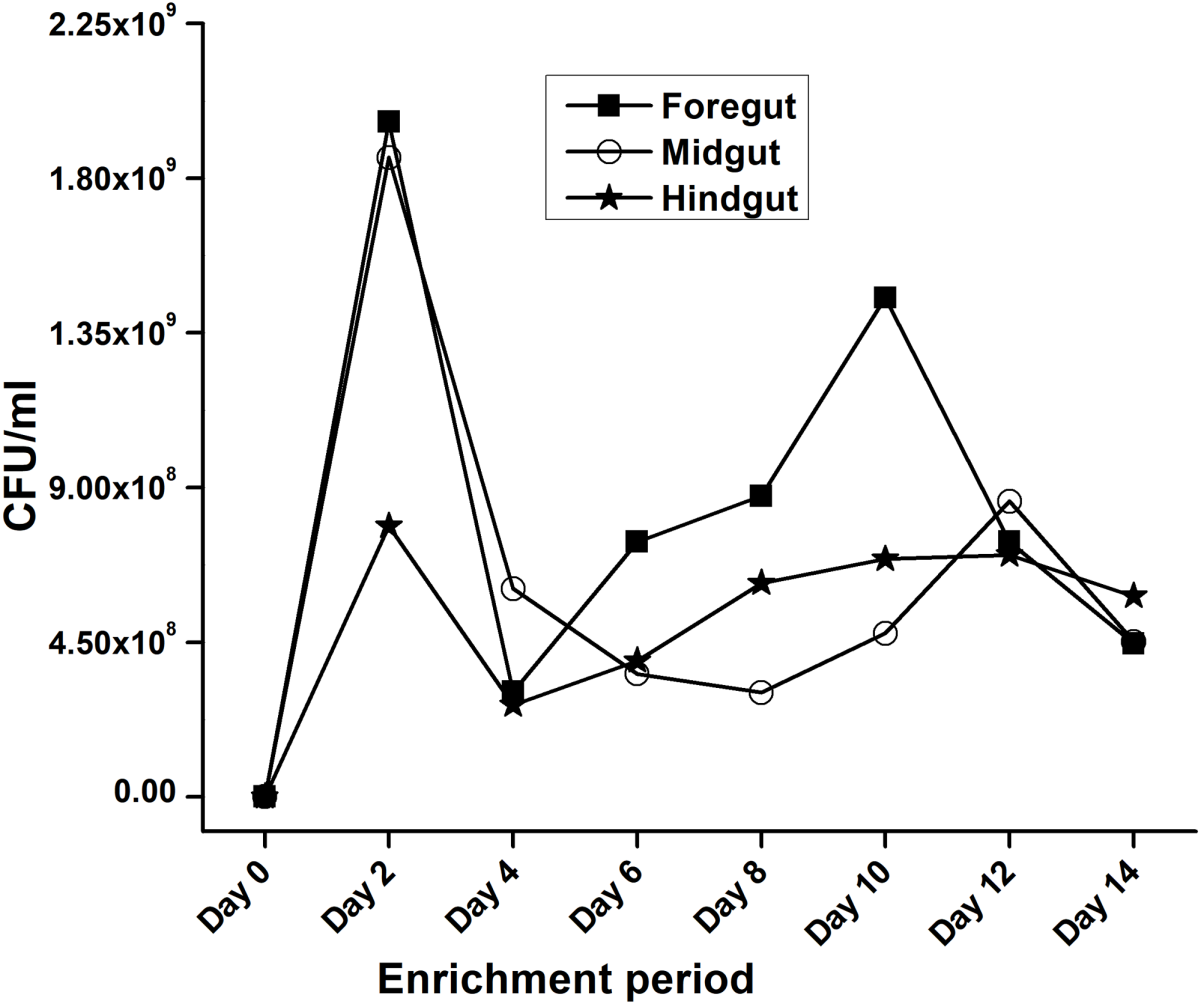
Progress of enrichment for lignocellulose degrading bacteria extracted from the gut regions of *H. armigera*.

**Figure 3:**
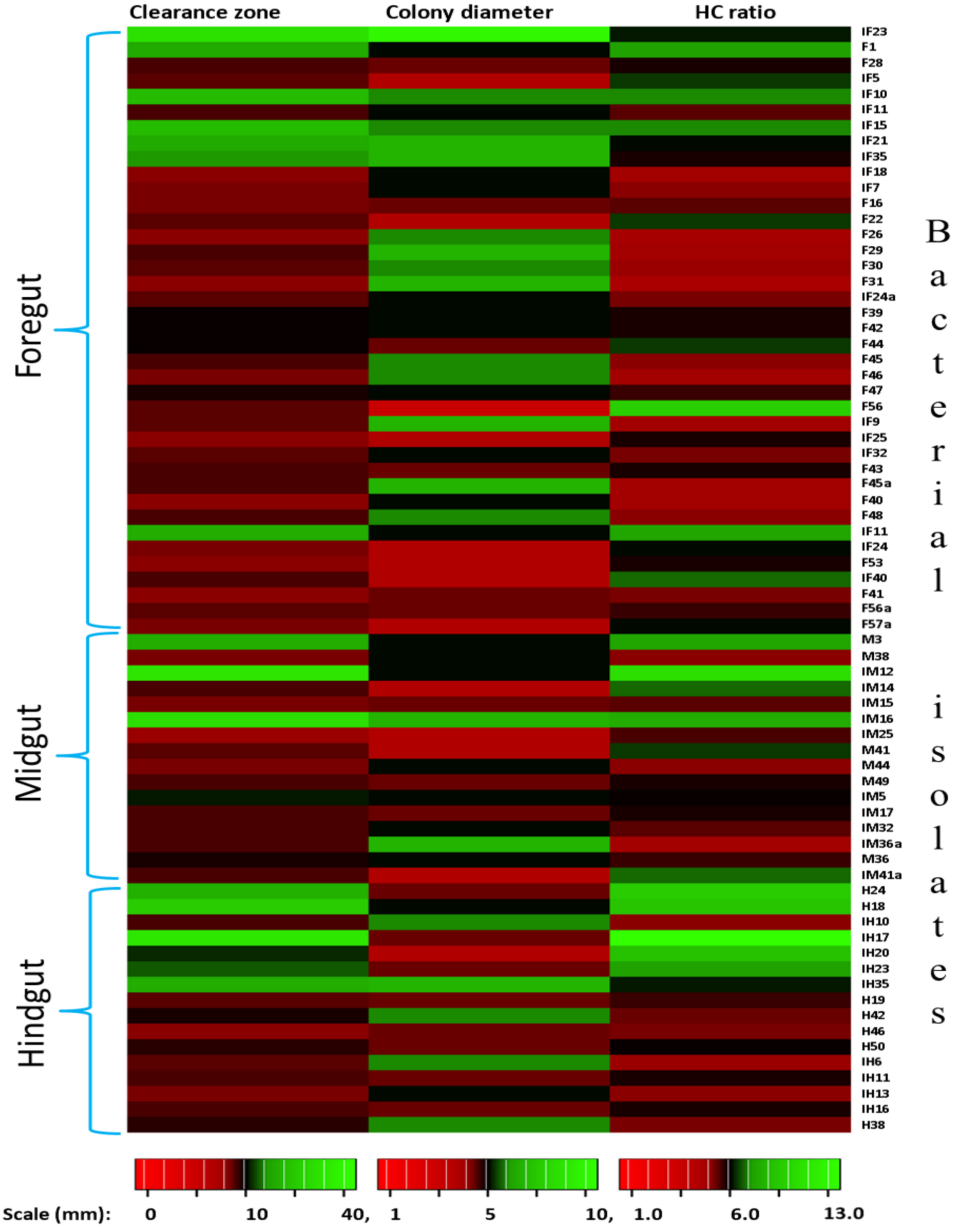
Heat map showing the Carboxymethyl cellulose degrading activity and hydrolytic capacity ratio of the enriched bacteria after isolation from different gut regions of *H. armigera*.

In the present study, monitoring the TVC during enrichment showed highest of 1. 96 ×10^9^colony forming units (CFU)/mL in FG on 2nd day while in MG and HG , TVCs were 1. 86 × 10^9^ and 0. 78 × 10^9^CFU/mL of extract, respectively (Fig. 2). In case of FG and HG, the lowest number of bacteria (3.06 and 2. 68 × 10^8^CFU/mL) were detected on 4th day of enrichment; while the lowest bacterial load from MG (3. 0 × 10^8^ CFU/mL extract) was attained on 8th day of enrichment. Among the three gut regions, the overall bacterial load was lowest in HG showing positive correlation with the digestive physiology of the insect as HG is largely responsible for assimilation of digested food. We observed that TVC of the CDB during enrichment followed an irregular trend of slight increase from 4th to 12th day and then declined again indicating reduced viability of the cells. Through enrichment process, several isolates with unique colony characteristics were isolated, purified, encoded and screened for cellulolytic activity. Initially, a total of 177 isolates from all three regions of the gut were isolated. These comprised 71, 55, and 51 isolates from FG, MG and HG respectively. Of these, most of the isolates did not grow on BMS-CMC agar media post 48 hrs of incubation at 37 °C. Finally, a total of 71 isolates showed noticeable growth on BMS agar plates containing 0.5% CMC (W/V) among which 39 bacteria were from FG while MG and HG each contributed 16 cellulase positive bacteria (Table 1). Among the total (71) cellulose positive bacteria, 46 isolates showed small or negligible clearance zones (<1 cm) on CMC-agar plates (Fig. 3). In all, 25 isolates comprising of 11 from FG while 5 and 7 from MG and HG, respectively showed discernible activities by displaying a significant zone of CMC clearance around the colonies indicating potential cellulolytic repertoire (Fig. S1A). These CDB exhibited diverse hydrolytic capacity ratios. The hydrolytic capacity ratio of the CDB from all three regions ranged from 1.0 to 12.67 with the highest ratio shown by HG isolates. Thirty-two and 45% of the CDB showed a HC between 0.5–2.0 and 2.1–4.0 respectively, while 9% of the isolates showed HC ratio of >6 cm (Fig. S1B). After screening, the potential cellulase positive isolates from all three gut regions were identified by using molecular approach employing 16S rDNA gene amplification and sequencing.

**Table 1:** Distribution and cellulose degrading potential of bacteria isolated from different regions of the gut of *H. armigera*. Values are represented as number of isolates followed by percentage (%) of total number of isolates in parenthesis.

**Gut region**	**Total no. of isolates**	**No. showing activity & (%)**	**No. showing low activity & (%)**	No. showing medium activity & (%)	**No. showing high activity & (%)**	**No. showing no activity & (%)**
Foregut	39	34 (87.18)	24 (61.53)	9 (23.07)	1 (2.56)	5 (12.82)
Midgut	16	13 (81.25)	7 (43.75)	4 (25)	2 (12.5)	3 (18.75)
Hindgut	16	12 (75)	3 (18.75)	8 (50)	1 (6.25)	4 (25)
**Total**	**71**	**59 (83.10)**	**34 (47.88)**	**21 (29.57)**	**4 (5.63)**	**12 (16.90)**

### Identification and phylogenetic analysis

**Figure 4:**
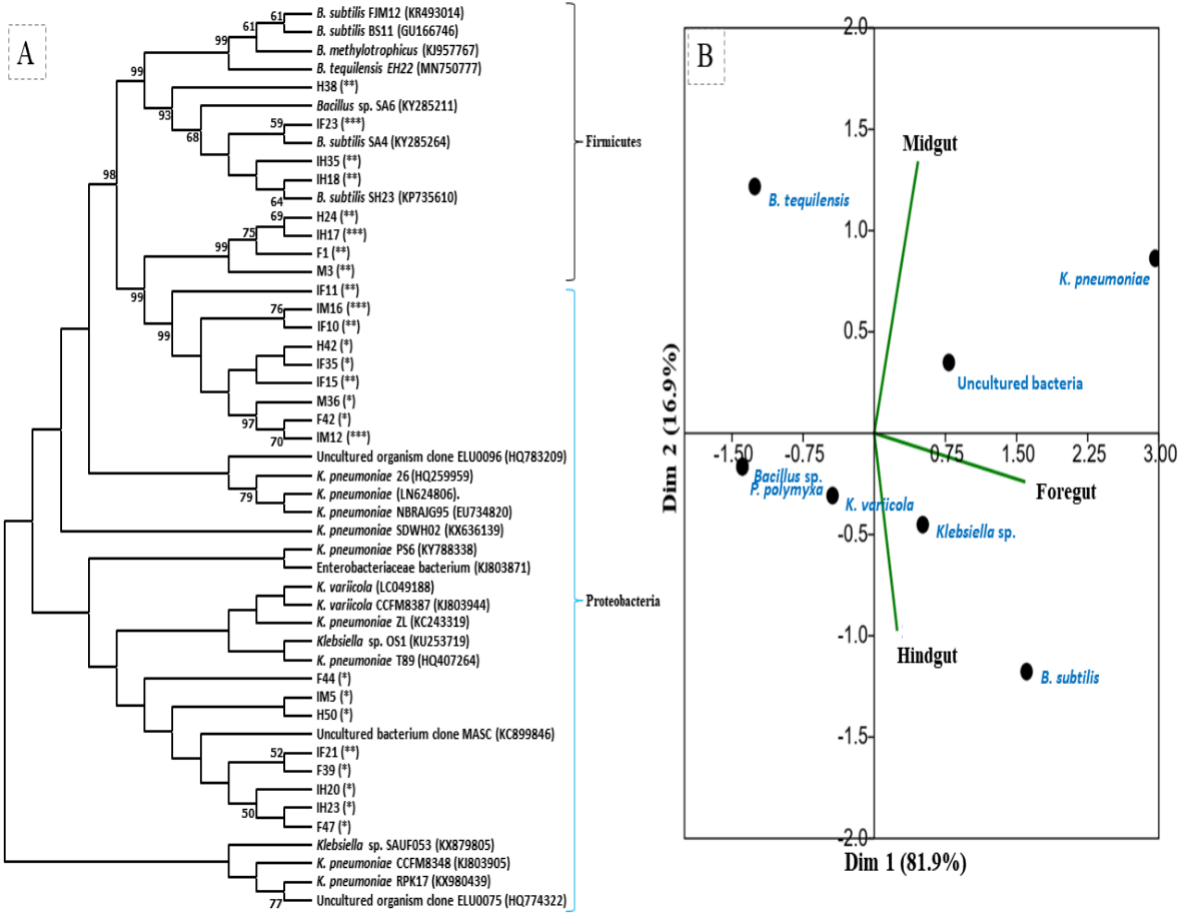
(A) Phylogenetic tree of potential cellulolytic bacteria isolated from the gut-systems of *H. armigera*. Bootstrap values are indicated at nodes. (B) PCA analysis of gut bacteria showing biplot of bacterial species and gut regions of cotton bollworm. The symbols in parenthesis indicate: *, low; **, medium, and ***, high activities of isolates.

**Figure 5:**
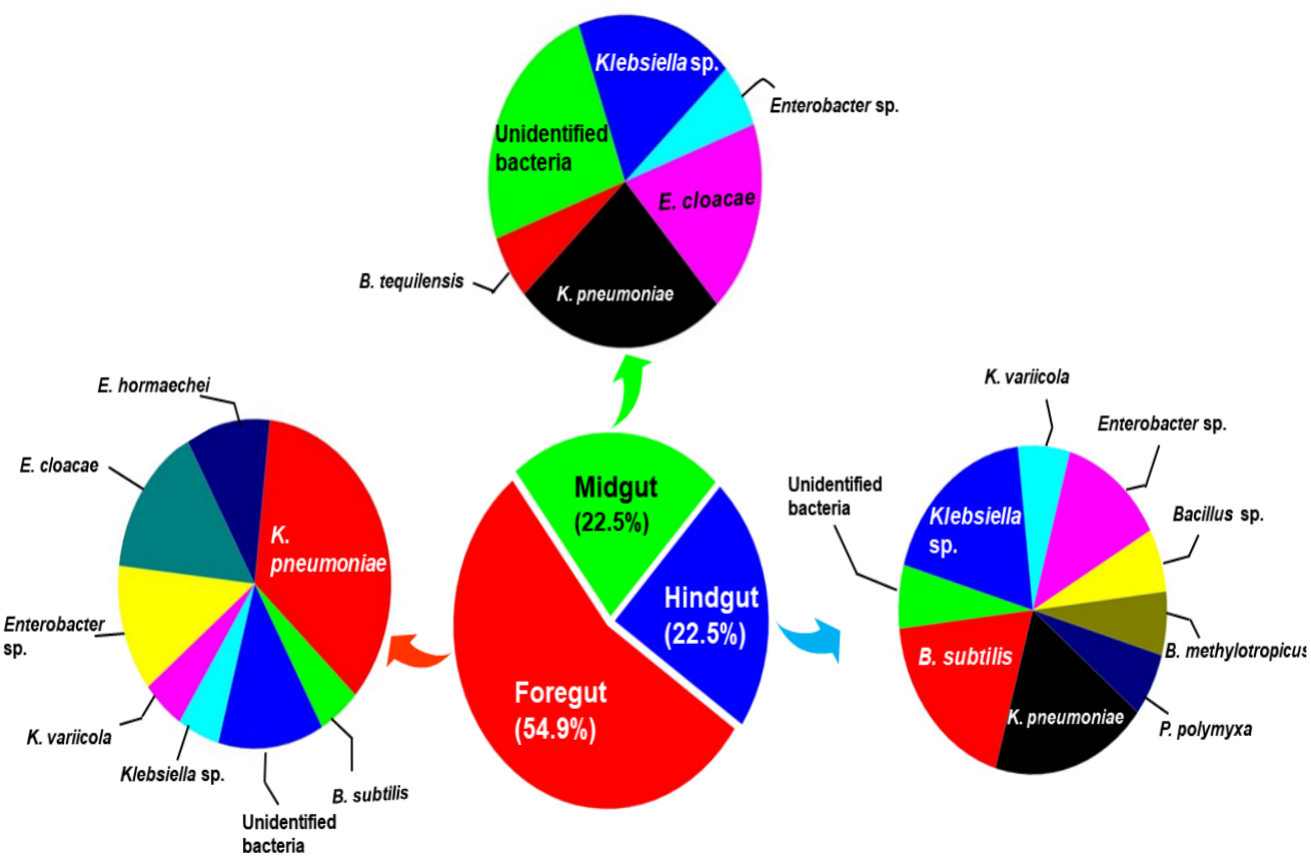
Relative abundance (%) and species richness of culturable bacterial species in different regions of the gut-system of *H. armigera*.

The amplification, sequencing and BLASTn search of 16S rRNA genes indicated that isolated bacteria belonged to 2 major phyla namely Firmicutes and *γ*-proteobacteria (Fig. 4A). Though many isolates shared similar habitat within the insect gut, the 16S rRNA sequencing affiliated them to different species of prokaryotes such as *Bacillus subtilis*, *B. methylotrophicus*, *B. tequilensis*, *Klebsiella pneumoniae*, *K. variicola*, *Enterobacter cloacae* and *E. hormachei* (Table 2). Amongst these, the members of genus *Klebsiella* were the most abundant and accounted for 39.43% of the total CDB in all three gut regions combined, whereas 11.26 and 21% isolates showed similarity to members of genera like *Enterobacter* and *Bacillus,* respectively (Fig. 5). In comparison, members of *Klebsiella* accounted for 35.89% in FG, while 43.75% each in MG and HG. Similarly, members of *Bacillus,* the second most abundant genus accounted for 5, 6 and 31% of total isolates enriched from FG, MG and HG, respectively. However, in case of FG and MG, members belonging to genus *Enterobacter* were abundant after *Klebsiella* showing 23 to 25% abundance respectively. The PCA analysis showed that strains like *Klebsiella* sp., *K. pneumoniae*, *B. Subtilis* and uncultured bacterium formed, principal component one (PC1) with a variance of 81.77% (Fig. 4B). However, the principal component two (PC2) which accounted for 16.97% variance was comprised of *B. tequilensis*, uncultured bacterium clones and *K. pneumoniae*. Similarly, the *B. subtilis*, *B. methylotrophicus*, *Paenibacillus polymyxa* and *K. pneumoniae* which formed PC3 showed least variance (1.23%). The PCA plots further suggested that *Klebsiella* sp. and *B. subtilis* dominated the FG and HG regions, whereas uncultured bacteria and *K. pneumoniae* were abundant in the MG region of the *H. armigera* larvae. The sequences obtained from 16S rDNA gene sequencing were submitted to the NCBI database under the accession numbers ranging from MT052333 to MT052357. After molecular identification and plate-based screening assay, 4 potential isolates, 2 each from genera, *Bacillus* and *Klebsiella* viz., *B. subtilis* IF23, *K. pneumoniae* IM12, *K. pneumoniae* IM16 and *B. subtilis* IH17 that showed larger zones of CMC clearance were selected further to check their cellulolytic repertoire.

**Table 2:** Likely genera and percent similarity of the potential activity showing isolates with the NCBI relative species determined by BLASTn search. Activity (Cm): Low (0.5–1.9); Medium (2.0–3.5); High (≥3.6).

**Isolate code**	**Accession** **no.**	**Likely genus**	**% Similarity**	**Activity**
F1	MT052342	*Bacillus subtilis* FJM12	97	Medium
IF10	MT052333	*Klebsiella pneumonia* NBRAJG95	99	Medium
IF11	MT052340	*Klebsiella pneumoniae* RPK17	98	Medium
IF15	MT052337	Uncultured organism clone ELU0096	98	Medium
IF21	MT052335	*Klebsiella pneumoniae* ZL	96	Medium
IF23	MT052343	*Bacillus subtilis* SA4	98	High
IF35	MT052341	Uncultured organism clone ELU0075	98	Low
F39	MT052339	*Klebsiella pneumoniae* T89	96	Low
F42	MT052338	*Klebsiella pneumoniae* SDWH02	100	Low
F44	MT052336	*Klebsiella* sp. OS1	98	Low
F47	MT052334	*Klebsiella variicola* CCFM8387	97	Low
M3	MT052344	*Bacillus tequilensis* EH22	100	Medium
IM12	MT052346	*Klebsiella pneumoniae*	99	High
IM5	MT052345	Uncultured bacterium clone MASC	99	Low
IM16	MT052347	*Klebsiella pneumoniae* 26	97	High
M36	MT052348	*Klebsiella pneumoniae* PS6	99	Low
IH17	MT052352	*Bacillus subtilis* FJM12	98	High
IH18	MT052355	*Bacillus subtilis* SH23	97	Medium
IH20	MT052356	*Klebsiella* sp. SAUF053	97	Low
IH23	MT052357	*Klebsiella variicola*	97	Low
H24	MT052353	*Bacillus methylotrophicus*	97	Medium
IH35	MT052351	*Bacillus* sp. SA6	96	Medium
H38	MT052354	*Bacillus subtilis* BS11	95	Medium
H42	MT052350	Enterobacteriaceae bacterium CCFM8314	98	Low
H50	MT052349	*Klebsiella pneumoniae* CCFM8348	96	Low

### Effect of substrate concentration and pH

**Figure 6:**
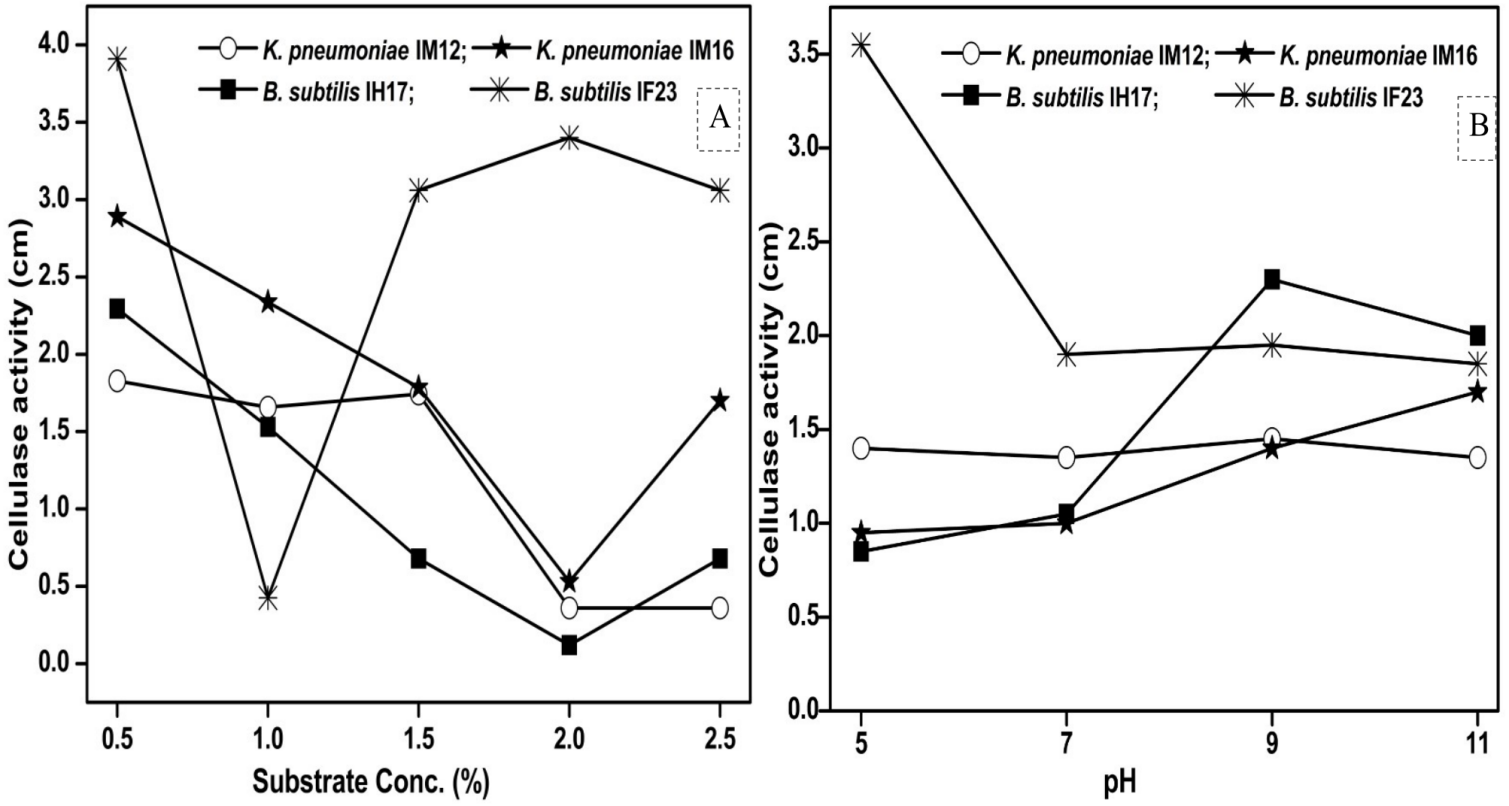
Effect of the concentration of CMC as substrate (A) and pH (B) on cellulose degrading activity of potential bacteria.

Since pH and concentration of the carbon source (substrate) are vital for enzyme production, we next studied how the variations in pH and substrate concentration in the culture media influence the cellulase activities of each selected isolate. We observed that the maximum concentration of CMC tolerated by *B. subtilis* IF23 was 2% however, the isolates preferred 0.5% CMC as they displayed higher zones of clearance in the range of 1.82 to 3.91 cm (Fig. 6A). Similarly, pH 5.0 was optimum for *B. subtilis* IF23 as it exhibited highest of 3.5 cm zone of CMC clearance. However, in case of *K. pneumoniae* IM12 and *B. subtilis* IH17, the optimum cellulose degradation was achieved at an alkaline pH, i.e, 9.0 (Fig. 6B). The optimum pH required for maximum cellulolytic activity by *K. pneumoniae* IM16 was 11.0 thus showing a large zone of substrate clearance measuring 1.7 cm in diameter.

### Determination of growth curve

The growth of the *B. subtilis* IF23 was initially slow which started after 20 hrs of incubation and continued till 96 hrs followed by a declining trend. Unlike *B. subtilis* IF23, the other isolates viz., *K. pneumoniae* IM12, *K. pneumoniae* IM16 and *B. subtilis* IH17 showed continuous growth up to 104 hrs (Fig. S2). In case of all the isolates, the exponential growth phase ranged from 48 to 120 hrs on CMC. In comparison, the growth of *K. pneumoniae* IM16 and *B. subtilis* IH17 were slow, irregular and extended up to 144 and 168 hrs respectively, which were followed by a declined pattern. Overall, the growth patterns of potential CDB in the present study demonstrated a positive correlation with enzyme activity and period of incubation. Among the tested isolates, higher and fast growth was observed for strain *K. pneumoniae* IM12, as it showed an absorbance as high as 2.17 @600 nm after 104 hrs of incubation. The extended growth pattern and higher absorbances shown by *K. Pneumoniae* IM12 makes this isolate potentially useful in some industrial processes which require longer durations of fermentation.

### Enzyme activities and principal component analysis

#### Endoglucanase activity

The selected cellulolytic isolates resulted the endoglucanase activity in the order as *B. subtilis* IH17> *B. subtilis* IF23> *Klebsiella pneumoniae* IM12> *K. pneumoniae* IM16. Filter paper was preferred as the most suitable substrate for endoglucanase productions by all tested isolates. The highest endoglucanase (CMCase) activity of 179.30 IU/mL extract was shown by *B. subtilis* IH17 on FP followed by *K. pneumoniae* IM16 (65.09 IU/mL extract) (Fig. S3A). Among the tested agro-wastes used as carbon sources, WH and CS were the least preferred substrates for endoglucanase activities. The overall substrate preference by the isolates followed a trend of FP>SCB>SD>GS>CMC>CS>WH (Fig. S4). The cumulative endoglucanase activity of all isolates on tested carbon sources was 630.52 ±2.8 IU/mL extract.

#### Xylanase activity

In the case of xylanase assay, highest activity of 158.78 IU/mL extract was produced on FP by *B. subtilis* IH17 whereas the lowest activity (43.92 IU/mL extract) was achieved with xylan as substrate by *K. pneumoniae* IM12 (Fig. S3B). Overall, the tested substrates for xylanase production performed in the order of merit as FP>SD>SCB>GS>xylan>WH>CS (Fig. S4) whereas, in case of all tested bacteria, the pattern of xylanase activities were similar to that observed for endoglucanase activity. Among the tested substrates, FP in comparison to other carbon sources was found most favored as all the tested isolates on FP resulted in higher enzyme production. Further, the xylanase activities of *B. subtilis* IF23 and *K. pneumoniae* IM12 on GS were 1.2 fold higher than their relative strains viz., *B. subtilis* IF17 and *K. pneumoniae* IM12 respectively. The bacterium, *K. pneumoniae* IM16 showed more preference towards FP followed by SD and depicted 157.7 and 121.96 IU/mL extract activities respectively. The endoglucanase and xylanase production by all isolates on agricultural wastes showed somewhat a similar pattern.

#### *β*-glucosidase activity

The highest of 269.14 IU/mL extract, *β*-glucosidase activity was produced by *B. subtilis* IF17 on SCB as substrate. Of all substrates tested, the higher activity was observed on SCB followed by SD, CMC, GS, FP, WH and CS (Fig. S4). The isolate *B. subtilis* IF23 showed higher activity (59.99 IU/mL extract) on SD while lowest of 16.67 IU/mL extract, activity was estimated on WH. In comparison, the *K. pneumoniae* strains IM12 and IM16 showed lower *β*-glucosidase activities (35.5 and 34.4 IU/mL extract respectively) than *B. subtilis* strains on all tested substrates. The potentially most active *B. subtilis* IH17 demonstrated more preference towards SCB followed by SD with an activity of 30.96 IU/mL extract; however, it showed least production of the enzyme on CS after the incubation period of 10 days (Fig. S3C). Comparatively, the overall *β*-glucosidase activity (248.0 IU/mL extract) produced by all tested isolates was low when compared with endoglucanase and exoglucanase activities.

#### Exoglucanase activity

The exoglucanase, also known as avicellase activity was observed maximum (46.53 IU/mL extract) on SCB as carbon source by *B. subtilis* IH17 which showed least preference towards GS substrate depicting only 2.62 IU/mL extract. *Bacillus subtilis* IF23 exhibited the highest activity of 9.89 IU/mL extract on FP while it showed lowest activity (3.43 IU/mL extract) on WH as substrate (Fig. S3D). In contrast, the *K. pneumoniae* strains (IM2 and IM16) showed better activities on GS and SD respectively depicting value of 6.40 and 8.45 IU/mL extract. The overall avicellase activity was found relatively less than the other enzyme activities under consideration. Among all enzyme activities determined, the endoglucanase and xylanase activities were much higher in all isolates than others, thereby describing the endo-cellulolytic or xylanolytic nature of these isolates. The exoglucanase activity on all substrates followed an order of SCB>FP>SD>Avicel>GS>WH and lowest on CS powder (Fig. S4).

**Figure 7:**
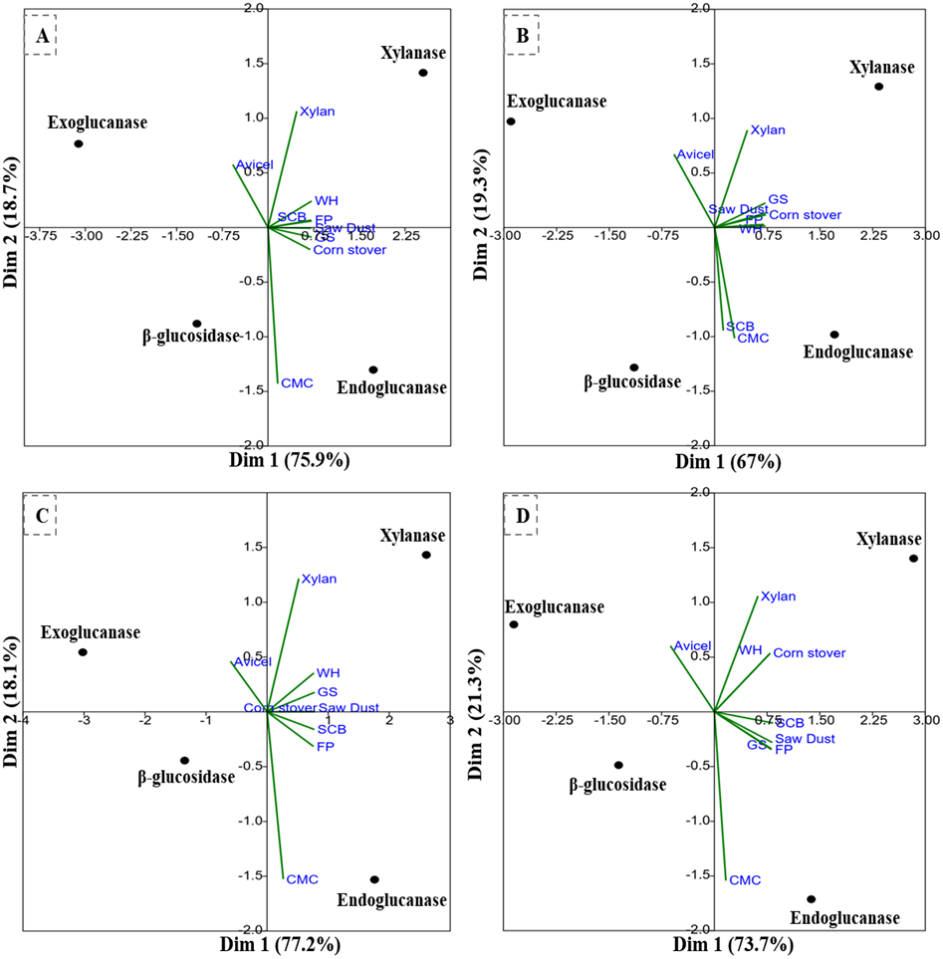
PCA biplots of the enzyme activities by potential isolates on different commercial and agro-wastes residues used as substrates. (A) *B. subtilis* IF23, (B) *B. subtilis* IH17 (C) *K. pneumoniae* IM12, and (D) *K. pneumoniae* IM16.

The PCA analysis of the multi-scale enzyme activity data demonstrated an obvious trend for enzyme production on different substrates. The PCA clustering of enzyme activities by *B. subtilis* IF23 accounted for 97.7% of the variability (81.3% and 16.4%) as shown in Fig. 7A. The PCA of enzyme activities produced by *B. subtilis* IF23 demonstrated the suitability of FP, CMC, GS, CS, SCB and SD as favorable substrates for endoglucanase production while xylanase was best produced on WH and xylan as substrates. In contrast, the bacterium, *B. subtilis* IH17 showed more xylanase activity on the substrates, viz., FP, GS, CS and SD with a variance of 86.3% (Fig. 7B). In case of all isolates, exoglucanase activity was attributed majorly to Avicel substrate. However, the PCA analysis of enzyme activities by *Klebsiella* strains such as *K. pneumoniae* IM12 and *K. pneumoniae* IM16 depicted the variance of 180.9% and 95% respectively (Figs. 7C and 7D). The *K. pneumoniae* IM12 showed more preference for xylanase activities with the tested substrates. However, it showed endoglucanase activity only on CMC, FP and SCB substrates (Fig. 7C). The PCA results showed that there was no obvious trend for *β*-glucosidase activities of the bacteria. The PCA of *B. subtilis* IF23 was significantly different than *B. subtilis* IH17 which showed higher production of xylanase on all agricultural wastes tested except SCB. However, both the *K. pneumoniae* strains (IM12 and IM16) shared similar patterns for enzyme production with minor variations. The multivariate analysis further explained that among the tested agro-wastes, WH was the best substrate for xylanase production while SCB significantly induced the secretion of endoglucanases by all potential bacterial strains.

## Discussion

Since insects represent the most diverse group of animals on earth, their success relies largely on their gut microflora that contributes to their diversity and fast adaptability to a wide range of environmental conditions. Insects are known to share co-speciation with their gut symbionts *(Hosokawa et al., 2006)*. However, the diversity of culturable lignocellulose hydrolyzing bacteria in different regions of the gut of *H. armigera* is largely unknown to date, despite few investigations that employed whole gut metagenomics (Gayatri et al., 2012; Shinde et al., 2019). Although these recent studies have reported the diversity of bacteria in the gut of *H. armigera*, none of these studies focused on the region-wise community structure of CDB. Using enrichment technique, the present research highlighted the gut-regions of *H. armigera* as potential reservoirs of lignocellulose hydrolyzing bacterial symbionts. Among the three gut regions, highest numbers (54%) of cellulolytic bacteria were isolated from FG region while in MG and HG, the numbers of cellulase positive isolates were nearly half of the former. The higher number of bacterial isolates observed in FG could be attributed to the fact that this gut region is the primary organ to receive ingested food contaminated with enormous number of bacteria from the environment *(Bignell, Roisin & Lo, 2011)*. The insect midgut is assumed as the primary site for digestion and absorptive processes *(Marzban, 2012)*. The few species of activity showing isolates in MG as compared to FG and HG could also be a consequence of highly alkaline pH and digestive milieu whereby majority of the enzymes are secreted endogenously in insects *(Ohkuma & Brune, 2011)*. Although the bacterial isolates of FG were higher than the other two gut sections, the activities of most of these isolates were relatively low. In comparison, the cellulolytic activities of the bacteria from HG region were higher where 31% of the isolates showed higher cellulolytic activity.

*Helicoverpa armigera* harbored a considerable variety of CDB across its gut-systems, where an uneven number of symbiotic bacterial species are present in different compartments of the gut. *Klebsiella* was found to be the most dominant genus across the gut-systems. After *Klebsiella*, many species of *Enterobacter* and *Bacillus* were observed as the dominant groups in FG and HG respectively. The observed cellulolytic bacterial community structure of *H. armigera* is in close agreement with the MG bacterial community of gypsy-moth larvae which encourages Firmicutes and *γ*-proteobacteria *(Broderick et al., 2004)*. In contrast to the previous study (Gayatri et al., 2012, only two species of *Enterobacter* were detected in the gut of *H. armigera* while the members of *Enterococcus* were completely absent. Further, when compared with other insects, the genera-wise composition of bacteria in *H. armigera* was different from the beetle, *P. ephipiata* in which 50% of MG bacteria belonged to phylum Actinobacteria and *Clostridia.* The authors also reported dominance of *Lactobacillales*, *Clostridiales*, and *Bacteroidetes (Egert et al., 2003)* that represented over 80% of the total bacterial load in HG. This variation in the community structure could be a consequence of dietary preferences of insects as the beetle predominantly feeds on hardwoods while *H. armigera* devours on tender parts of the plant. The ubiquitous presence of the members of enteric gram-negative *Klebsiella* and gram-positive *B. subtilis* in the *H. armigera* comprises a constant fraction of gut microbiota thus these species can be regarded as part of its core bacteria. These core bacteria contribute to the host fitness due to their role in carbohydrate hydrolysis and fermentation besides nitrogen metabolism, vitamin and pheromone productions (Rizzi et al., 2013). The presence of cellulolytic bacteria, such as *B. subtilis* and *Klebsiella* spp. have been established previously in other lepidopterans viz., silkworm (Anand et al., 2010, *Diatrea saccharalis (Dantur et al., 2015)*, etc. Similarly, the existence of *Bacillus* and *Klebsiella* species which are predominantly active towards LC degradation, signposts their functional implications and contribution to digestion of plant matter within the insect host (Gayatri et al., 2012).

The growth patterns of the potential isolates were slow in contrast to *B. subtilis* BY-2 (Yang et al., 2014) reaching a logarithmic phase after 44 h and continued up to 160 h except for *K. pneumoniae* IM12 which started to decline only after 120 hrs. The strain, *B. subtilis* IF23 showed a longer lag phase than other tested strains which could be a consequence of adaptations to growth medium and culture conditions by the bacterium. However, the extended exponential phase on CMC media also indicates the inducible nature of the bacteria suitable for industrial purposes. The extended growth phases of potential bacteria were also longer than that of *B. licheniformis* which has been isolated previously from biosulphidogenic bioreactor *(Van-Dyk et al., 2009)* and decaying wood (Damiano, 2003).

The slow degradation of agro-biomass caused due to recalcitrant nature of LC, end-product inhibition and enzyme inactivation necessitates the employment of a cocktail of efficiently acting enzymes possessing synergistic mechanism for successful biomass conversion. In this regard, the potential bacteria were further characterized for the production of three cellulases (endoglucanase-EC3.2.1.4, exoglucanase-EC3.2.1.91, *β*-glucosidase-EC3.2.1.21) together with xylanase on various LC waste materials. Furthermore, for large data sets like ours involving multiple variables, PCA is the method of choice that reduces the ambiguity while maintains the original observations *(Sadalage et al., 2020).* Since PCA clustering helps to scale multi-variable data sets and can identify significant components *(Joliffe & Morgan, 1992)* we performed PCA to characterize correlations of different enzyme activities produced by potential bacteria on various substrates (Fig. 7). The linear transformation of data variables to principal components provided a clear view of the substrates responsible for production of specific enzymes. The PCA analysis of the data suggested that potential isolates can secrete higher amounts of endoglucanase and xylanase enzymes but not exoglucanase and *β*-glucosidases. Apart from the commercially available CMC substrate, the PCA analysis signified SCB as a preferred substrate for secretion of endoglucanases by all tested isolates. Similarly, in addition to FP as substrate, WH was found as a good inducer for xylanase secretion in potential isolates under consideration. The PCA analysis further depicted that avicellase activity was marginal than other tested enzyme activities in all isolates. Among the tested agro-wastes, SCB, GS, SD, and CS were preferred as substrates for endoglucanase production by *B. subtilis* IF23 while WH was found most important for the yield of xylanase enzyme by all potential bacteria. This enzymatic plasticity of potential isolates on different substrates could be largely due to the composition and nature of carbon sources under consideration *(Bledzki, Mamun & Volk, 2010)*. Further probable reason for the differential production of these enzymes could also be attributed to the genotype or specific metabolic activities of the bacteria.

The endoglucanase activity of potential isolates was higher than the activity of *Bacillus* and *Klebsiella* strains isolated from the gut of *Reticulitermes speratus (Cho et al., 2010)*. Although the maximum exoglucanase activity shown by *B. subtilis* strains IF23 and IH17 were higher than *Bacillus* species *(Cho et al., 2010)*, the activities of *Klebsiella* strains in both the studies were nearly in similar order. In contrast, the exoglucanase activity was very low when compared with *Bacillus circulans* and *K. pneumoniae* isolated from 5th instar larvae of *Bombyx mori (Anand et al., 2010)*. Interestingly, our isolates also possessed the capability to produce *β*-glucosidase on all tested agro-waste substrates. The *β*-glucosidase activity by *B. subtilis* IH17 was manifold higher than the activity of *K. pneumoniae* and *Bacillus* sp. (2.1 and 2.5 IU/ml respectively) isolated from the termite gut *(Cho et al., 2010)*. The *Bacillus* species are well-known candidates for many industrial processes such as *B. subtilis* secretes multi-enzyme mini-cellulases employed for LC transformation. Taken together, the findings indicate the tremendous potential of insect gut bacteria that can be exploited for several biotechnological applications after thorough understanding of the involved mechanisms. The potential degradation of cellulosic wastes by *K. pneumoniae* and *B. subtilis* strains further advocate their symbiotic roles for *H. armigera* to enhance its food utilization efficiency *(Xia et al., 2017)*.

The symbiotic gut bacteria of insects are known for their significant impact on physiological activities of the host *(Shinde et al., 2019)*. In insects, including *H. armigera*, it is generally assumed that enzymes derived from bacterial symbionts provide an auxiliary role during adaptation to different diets *(Genta et al., 2006)*. This ability is due to the inherent capacity of gut symbionts to adjust with varying diets through induction mechanisms or some changes in bacterial populations, allowing the expression of one community over others *(Kaufman & Klug, 1991; Santo-Domingo et al., 1998)*. The gut system of *H. armigera* is a complex holobiont that represents a prosperous reservoir of many unique and novel lignocellulase encoding genes. Additionally, the diverse microbial communities with varying metabolic capabilities might have important physiological implications for the host particularly in fast adaptation to different habitats and feeding patterns.

Contrary to previous metagenomic studies where authors have reported enormous diversity of bacteria in insect guts, we recovered eight species of bacteria from the gut-regions of *H. armigera*. Primarily the gut bacterial communities of *H. armigera* are highly dynamic in nature, shifting in response to time, location and dietary preferences *(Paramasiva et al., 2014)*. The further possible causes for lower diversity of bacterial isolates could be attributed to two main reasons. First, the main goal of present research was isolation of LC degrading bacteria through enrichment on CMC which restricted the growth of non-cellulolytic bacteria. Second, the isolated bacteria were cultured under aerobic conditions which could have further delimited anaerobic bacteria as the insect gut is mostly anoxic in nature. Hence, further research is needed to explore the diversity of unculturable and anaerobic CDB from the individual gut-regions of *H. armigera* by employing culture-independent approaches of metagenomics. Overall, these culture-based and culture-independent approaches may potentially provide a holistic view of microbiome structure and their functions at multiple layers within the gut of *H. armigera*.

## Conclusion

Although the presence of bacteria has been previously reported in *H. armigera*, none of the studies have focused on CDB from individual gut regions of this disaster causing pest species. In this aspect, our efforts have elaborated the profile of CDB from the individual gut-sections of *H. armigera*. Though FG harbors a considerable variety of bacteria, their cellulolytic activities were very low when compared with HG isolates. Among the observed bacteria, members of genus, *Klebsiella* were dominant throughout the gut-systems. The characterization of the potential CDB revealed their cellulolytic repertoire by hydrolyzing various agro-wastes. The tested isolates further showed varying capability to secrete various lignocellulose degrading enzymes on SCB, FP, GS and SD indicating their suitability for bioconversion processes. The hydrolytic capacity of the isolates also supports the notion that symbiotic bacteria may possibly act synergistically with the host’s endogenous enzyme systems to break down lignocellulose consumed by the insect. Prospectively, the gut-system of *H. armigera* can serve as a potential reservoir for the bioprospection of lignocellulolytic bacteria/genes that can be harnessed for various bioconversion technologies to produce added-value products from LCB. Considering the vast diversity of lepidopterans (180,000 species), together with their exclusively phytophagous nature, the present work projects towards unraveling the treasure-trove of novel bacterial phylotypes which might have many biotechnological and industrial applications.

##  Supplemental Information

10.7717/peerj.11254/supp-1 Supplemental Information 1 Growth curve determination data. Growth pattern of selected potential cellulose degrading bacteria in BMS medium supplemented with 0.5% CMC as substrate.

10.7717/peerj.11254/supp-2 Supplemental Information 2 Raw data for HC ratio: Hydrolytic capacity of bacteria

10.7717/peerj.11254/supp-3 Supplemental Information 3 FASTA sequences.

10.7717/peerj.11254/supp-4 Supplemental Information 4 Total viable count calculation.

10.7717/peerj.11254/supp-5 Supplemental Information 5 Raw data: effect of Substrate concentration & pH on cellulase activity

## Additional Information and Declarations

### Field Study Permissions

The following information was supplied relating to field study approvals (i.e., approving body and any reference numbers): The co-author Ms. Afrin F. Shaikh had good contact with local farmers, Abdul Nabi ChandSaheb Shaikh and Mane Gajanan, who allowed us to visit their fields and collect insect samples.

### DNA Deposition

The following information was supplied regarding the deposition of DNA sequences: The sequences are available at GenBank: MT052333 to MT052357.

### Data Availability

The following information was supplied regarding data availability: The raw measurements are available in the Supplemental Files.

## References

[ref-1] Anand AAP, Vennison SJ, Sankar SG, Prabhu DIG, Vasan PT, Raghuraman T, Geoffrey CJ, Vendan SE (2010). Isolation and characterization of bacteria from the gut of *Bombyx mori* that degrade cellulose, xylan, pectin and starch and their impact on digestion. Journal of Insect Science.

[ref-2] Auer L, Lazuka A, Sillam-Dussès D, Miambi E, O’Donohue M, Hernandez-Raquet G (2017). Uncovering the potential of termite gut microbiome for lignocellulose bioconversion in anaerobic batch bioreactors. Frontiers in Microbiology.

[ref-3] Bignell DE, Roisin Y, Lo N (2011). Biology of termites: a modern synthesis.

[ref-4] Bledzki AK, Mamun AA, Volk J (2010). Physical, chemical and surface properties of wheat husk, rye husk and soft wood and their polypropylene composites. Composites: Part A.

[ref-5] Breznak JA, Brune A (1994). Role of microorganisms in the digestion of lignocellulose by termites. Annual Reviews in Entomology.

[ref-6] Broderick NA, Raffa KF, Goodman RM, Handelsman J (2004). Census of the bacterial community of the gypsy moth larval midgut by using culturing and culture-independent methods. Applied and Environmental Microbiology.

[ref-7] Brune A, Carsten D (2015). The gut microbiota of termites: digesting the diversity in the light of ecology and evolution. Annual Review of Microbiology.

[ref-8] Brune A, Miambi E, Breznak JA (1995). Roles of oxygen and the intestinal microflora in the metabolism of lignin derived phenyl propanoids and other monoaromatic compounds by termites. Applied and Environmental Microbiology.

[ref-9] Calusinka M, Marynowska M, Bertucci M, Untereiner B, Klimek D, Goux X, Sillam-Dussès D, Gawron P, Halder R, Wilmes P, Ferrer P, Gerin P, Roisin Y, Delfosse P (2020). Integrative omics analysis of the termite gut system adaptation to *Miscanthus* diet identifies lignocellulose degradation enzymes. Communications Biology.

[ref-10] Cho MJ, Kim HY, Shin K, Kim YK, Kim YS, Kim TJ (2010). Symbiotic adaptation of bacteria in the gut of *Reticulitermes speratus*: low endo-b-1, 4-glucanase activity. Biochemical and Biophysical Research Communications.

[ref-11] Damiano VB (2003). Application of crude xylanase from *Bacillus licheniformis* 77-2 to the bleaching of eucalyptus kraft pulp. World Journal of Microbiology and Biotechnology.

[ref-12] Dantur KI, Enrique R, Welin B, Castagnaro AP (2015). Isolation of cellulolytic bacteria from the intestine of *Diatraea saccharalis* larvae and evaluation of their capacity to degrade sugarcane biomass. AMB Express.

[ref-13] Dar MA, Pawar KD, Jadhav JP, Pandit RS (2015). Isolation of cellulolytic bacteria from the gastro- intestinal tract of *Achatina fulica* (Gastropoda: pulmonata) and their evaluation for cellulose biodegradation. International Biodeterioration and Biodegradation.

[ref-14] Dar MA, Pawar KD, Pandit RS (2018). Prospecting the gut fluid of giant African land snail, *Achatina fulica* for cellulose degrading bacteria. International Biodeterioration and Biodegradation.

[ref-15] Dar MA, Shaikh AA, Pawar KD, Pandit RS (2018). Exploring the gut of *Helicoverpa armigera* for cellulose degrading bacteria and evaluation of a potential strain for lignocellulosic biomass deconstruction. Process Biochemistry.

[ref-16] Dillon RJ, Dillon VM (2004). The gut bacteria of insects: nonpathogenic interactions. Annual Reviews in Entomology.

[ref-17] Duplouy A, Hornett EA (2018). Uncovering the hidden players in Lepidoptera biology: the heritable microbial endosymbionts.

[ref-18] Egert M, Wagner B, Lemke T, Brune A, Friedrich MW (2003). Microbial community structure in midgut and hindgut of the humus-feeding larva of *Pachnoda ephippiata* (Coleoptera: Scarabaeidae). Applied and Environmental Microbiology.

[ref-19] Engel P, Moran NA (2013). The gut microbiota of insects diversity in structure and function. FEMS Microbiology Reviews.

[ref-20] Fitt GP (1989). The ecology of *Heliothis species* in relation to agroecosystems. Annual Review of Entomology.

[ref-21] Gayatri PN, Ojha A, Kajla MK, Raj A, Rajagopal R Host plant induced variation in gut bacteria of *Helicoverpa armigera*. PLOS ONE.

[ref-22] Geng A, Cheng Y, Wang Y, Zhu D, Le Y, Wu J, Xie R, Yuan JS, Sun JZ (2018). Transcriptome analysis of the digestive system of a wood-feeding termite (*Coptotermes formosanus*) revealed a unique mechanism for effective biomass degradation. Biotechnology for Biofuels.

[ref-23] Genta FA, Dillon RJ, Terra WR, Ferreira C (2006). Potential role for gut microbiota in cell wall digestion and glucoside detoxification in *Tenebrio molitor* larvae. Journal of Insect Physiology.

[ref-24] Hammer Ø, Harper DAT, Ryan PD (2001). PAST: paleontological statistics software package for education and data analysis. Palaeontologia Electronica.

[ref-25] Hendricks CW, Doyle JD, Hugley B (1995). A new solid medium for enumerating cellulose-utilizing bacteria in soil. Applied and Environmental Microbiology.

[ref-26] Hosokawa T, Kikuchi Y, Nikoh N, Shimada M, Fukatsu T (2006). Strict host-symbiont co-speciation and reductive genome evolution in insect gut bacteria. PLOS Biology.

[ref-27] Joliffe IT, Morgan B (1992). Principal component analysis and exploratory factor analysis. Statistical Methods in Medical Research.

[ref-28] Kasana RC, Salwan R, Dhar H, Dutt S, Gulati A (2008). A rapid and easy method for the detection of microbial cellulases on agar plates using Gram’s iodine. Current Microbiology.

[ref-29] Kaufman MG, Klug MJ (1991). The contribution of hindgut bacteria to dietary carbohydrate utilization by crickets (Orthoptera, Gryllidae). Comparative Biochemistry and Physiology A.

[ref-30] Lemke T, Ulrich S, Egert M, Friedrich MW, Brune A (2003). Physicochemical conditions and microbial activities in the highly alkaline gut of the humus-feeding larva of *Pachnoda ephippiata* (Coleoptera: Scarabaeidae). Applied and Environmental Microbiology.

[ref-31] Lowry OH, Rosebrough NJ, Farri AL, Randall RJ (1951). Protein measurement with the folin phenol reagent. Journal of Biological Chemistry.

[ref-32] Luo C, Li Y, Liao H, Yang Y (2018). De novo transcriptome assembly of the bamboo snout beetle *Cyrtotrachelus buqueti* reveals ability to degrade lignocellulose of bamboo feedstock. Biotechnology for Biofuels.

[ref-33] Marzban R (2012). Midgut pH profile and energy differences in lipid, protein and glycogen metabolism of *Bacillus thuringiensis* cry1ac toxin and cypovirus-infected *Helicoverpa armigera* (Hübner) (Lepidoptera: Noctuidae). Journal of the Entomological Research Society.

[ref-34] Miller GL (1959). Use of dinitrosalicylic acid reagent for determination of reducing sugar. Analytical Chemistry.

[ref-35] Mitter C, Davis DR, Cummings MP (2017). Phylogeny and evolution of Lepidoptera. Annual Reviews in Entomology.

[ref-36] Ohkuma M, Brune A, Bignell DE, Roisin Y, Lo N (2011). Diversity, structure and evolution of the termite gut microbial community. Biology of termites: a modern synthesis.

[ref-37] Paramasiva I, Shouche Y, Kulkarni GJ, Krishnayya PV, Akbar SM, Sharma HC (2014). Diversity in gut microflora of *Helicoverpa armigera* populations from different regions in relation to biological activity of *Bacillus thuringiensisδ*-endotoxin Cry1Ac. Archives of Insect Biochemistry and Physiology.

[ref-38] Rizzi A, Crotti E, Borruso L, Jucker C, Lupi D, Colombo M, Daffonchio D (2013). Characterization of the bacterial community associated with larvae and adults of *Anoplophora chinensis* collected in Italy by culture and culture-independent methods. BioMed Research International.

[ref-39] Sadalage PS, Dar MA, Chavan AR, Pawar KD (2020). Formulation of synthetic bacterial consortia and their evaluation by principal component analysis for lignocellulose rich biomass degradation. Renewable Energy.

[ref-40] Santo-Domingo JW, Kaufman MG, Klug MJ, Holben WE, Haris D, Tiedje JM (1998). Influence on diet on the structure and function of the bacterial hindgut community of crickets. Molecular Ecology.

[ref-41] Shinde AA, Shaikh FK, Gadge PP, Padul MV, Govindwar SP, Kachole MS (2019). Conserved nature of *Helicoverpa armigera* gut bacterial flora on different host plants and in vitro interactions with PI proteins advocates role in host digestive physiology. Journal of the Saudi Society of Agricultural Sciences.

[ref-42] Sree KS, Varma A (2015). Biocontrol of Lepidopteran pests: use of soil microbes and their metabolites.

[ref-43] Sun JZ, Ding SY, Doran-Peterson J (2014). Biological conversion of biomass for fuels and chemicals: explorations from natural utilization systems. Royal society of chemistry energy and environment series.

[ref-44] Sun JZ, Scharf ME (2010). Exploring and integrating cellulolytic systems of insects to advance biofuel technology. Insect Science.

[ref-45] Tamura K, Stecher G, Peterson D, Filipski A, Kumar S (2013). MEGA6: molecular evolutionary genetics analysis version 6.0. Molecular Biology and Evolution.

[ref-46] Thompson JD, Gibson TJ, Plewniak F, Jeanmougin F, Higgins DG (1997). The CLUSTAL X windows interface: flexible strategies for multiple sequence alignment aided by quality analysis tools. Nucleic Acids Research.

[ref-47] Tokuda G, Watanabe H, Matsumoto T, Noda H (1997). Cellulose digestion in the wood-eating higher termite, *Nasutitermes takasagoensis* (shiraki): distribution of cellulases and properties of endo-*β*-1, 4-gIucanase. Zoological Science.

[ref-48] Tsegaye B, Balomajumder C, Roy P (2018a). Biodegradation of wheat straw by *Ochrobactrum oryzae* BMP03 and *Bacillus* sp. BMP01 bacteria to enhance biofuel production by increasing total reducing sugars yield. Environmental Science and Pollution Research.

[ref-49] Tsegaye B, Balomajumder C, Roy P (2018b). Biodelignification and hydrolysis of rice straw by novel bacteria isolated from wood feeding termite. 3 Biotech.

[ref-50] Tsegaye B, Balomajumder C, Roy P (2019a). Microbial delignification and hydrolysis of lignocellulosic biomass to enhance biofuel production: an overview and future prospect. Bulletin of the National Research Centre.

[ref-51] Tsegaye B, Balomajumder C, Roy P (2019b). Isolation and characterization of novel lignolytic, cellulolytic, and hemicellulolytic bacteria from wood-feeding termite *Cryptotermes brevis*. International Microbiology.

[ref-52] Ulyshen MD (2016). Wood decomposition as influenced by invertebrates. Biological Reviews.

[ref-53] Van-Dyk JS, Sakka M, Sakka K, Pletschke BI (2009). The cellulolytic and hemicellulolytic system of *Bacillus licheniformis* SVD1 and the evidence for production of a large multi-enzyme complex. Enzyme and Microbial Technology.

[ref-54] Watanabe H, Tokuda G (2001). Animal cellulases. Cellular and Molecular Life Sciences.

[ref-55] Weisburg WG, Barns SM, Pelletier DA, Lane DJ (1991). 16S ribosomal DNA amplification for phylogenetic study. Journal of Bacteriology.

[ref-56] Wilson DB, Irwin D (1999). Genetics and properties of cellulases. Recent progress in bioconversion of lignocellulosics (eds. G.T. Tsao, et al.). Advances in Biochemical Engineering/Biotechnology.

[ref-57] Xia X, Gurr GM, Vasseur L, Zheng D, Zhong H, Qin B, Lin J, Wang Y, Song F, Li Y, Lin H, You M (2017). Metagenomic sequencing of diamondback moth gut microbiome unveils key holobiont adaptations for herbivory. Frontiers in Microbiology.

[ref-58] Xie SX, Syrenne R, Sun S, Yuan JS (2014). Exploration of natural biomass utilization systems (NBUS) for advanced biofuel- from systems biology to synthetic design. Current Opinion in Biotechnology.

[ref-59] Yang W, Meng F, Peng J, Han P, Fang F, Ma L, Cao B (2014). Isolation and identification of a cellulolytic bacterium from the Tibetan pig’s intestine and investigation of its cellulase production. Electronic Journal of Biotechnology.

